# Kinematic and Radiographic Evaluation of Acromioclavicular Reconstruction with a Synthetic Ligament

**DOI:** 10.1155/2022/7144209

**Published:** 2022-05-28

**Authors:** Rafael F. Escamilla, Chad Poage, Scott Brotherton, Toran D. MacLeod, Charles Leddon, James R. Andrews

**Affiliations:** ^1^Department of Physical Therapy, California State University, Sacramento, CA, USA; ^2^Andrews Research and Education Foundation, Gulf Breeze, FL, USA; ^3^Andrews Institute for Orthopaedics & Sports Medicine, Gulf Breeze, FL, USA; ^4^Orthopedic Surgery & Sports Medicine, Palm Harbor, FL, USA

## Abstract

**Purpose:**

The optimal surgical technique for unstable acromioclavicular (AC) and coracoclavicular (CC) joint injuries has not yet been established. The biomechanical and radiographic effect of the LockDown device, a synthetic ligament for AC joint reconstruction, was evaluated to assess the optimal surgical technique for unstable AC and CC joint injuries. It was hypothesized that the LockDown device would restore AC joint kinematics and radiographic stability to near native values.

**Methods:**

Three fresh frozen cadaveric torsos (6 shoulders) modelled CC joint motion in their “native,” “severed,” and “reconstructed” states. The effects of stressed and unstressed native, severed, and reconstructed conditions on AC separation and CC distances in anteroposterior, mediolateral, and inferosuperior directions during shoulder abduction, flexion, and scaption were assessed. The analysis of variance (*p*, 0.05) was used to compare CC distance and peak AC distance in anteroposterior, mediolateral, and inferosuperior directions during shoulder flexion, abduction, and scaption measurements among native, severed, and reconstructed states with unstressed and stressed Zanca radiographic views.

**Results:**

From radiographic analyses, the CC distance was significantly greater (*p*=0.001) across the surgical state in stressed versus unstressed views. Mean difference between stressed and unstressed views was 1.8 mm in native state, 4.1 mm in severed state, and 0.9 mm in reconstructed state. The CC distance was significantly greater in the “severed” state (10.4 mm unstressed; 14.5 mm stressed) compared to the “native” state (*p*=0.016) (6.5 mm unstressed; 8.3 mm stressed) and compared to the “reconstructed” state (*p*=0.005) (3.1 mm unstressed; 4.0 mm stressed) and significantly less (*p*=0.008) in the “reconstructed” state compared to the “native” state. CC distances decreased from native to reconstructed, an average of 3.3 mm for unstressed and 4.3 mm for stressed. On average, peak AC joint separation distance in anteroposterior, mediolateral, and inferosuperior directions during shoulder-abduction, flexion, and scaption was shown to be restored to 11.5 mm of native values after reconstruction with LockDown device.

**Conclusion:**

Reconstruction of AC joint with LockDown synthetic ligament restores motion of clavicle and acromion to near native values, thereby decreasing scapular dyskinesis and enhancing AC joint stability.

## 1. Introduction

Injury to the acromioclavicular (AC) joint is a common occurrence, with joint dislocation accounting for 9% of all traumatic shoulder injuries [1]. Both the AC and coracoclavicular (CC) structures form a bridge between the clavicle and the scapula, acting as a stabilizer during shoulder motion through ligamentous and muscular attachments. High-grade injuries to the AC joint and the AC and CC ligaments, which are the two most commonly injured ligaments with AC injuries, result in abnormal motion between the clavicle and scapula [2]. This uncoupling effect destabilizes the shoulder girdle and results in a protracted scapula, which can lead to deleterious effects such as scapular dyskinesia, as well as altered glenohumeral biomechanics and loss of strength [3]. When treating injuries to the AC joint, it is vital to have a full understanding of this anatomic relationship and how it correlates to function.

Most clinicians agree that the optimal treatment for high grade injuries to AC joint is surgical intervention [4]. The optimal technique choice, however, remains a debate. Multiple techniques have been described, including joint pinning [5], hook plates [6], CC cerclages [7], endobuttons [8], ligament transfers [9], and repair of the native AC and CC ligaments [10]. Despite the availability of techniques and focus in the literature, the risk of significant complication still exists [11–13].

A systematic review of the literature by Woodmass et al. [14] analyzed complications following AC reconstruction and found that the rate of hardware irritation or residual pain was 26.7%, fracture rate was 5.3%, and loss of AC joint reduction was 26.8%. Loss of reduction postoperatively may be related to failure to restore the native anatomy and joint kinematics. An ideal surgical technique would address both anatomic and kinematic components while providing enough stability to keep reduction intact until soft tissue healing occurs.

**Figure 1:**
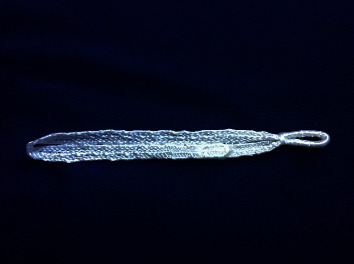
The lockdown device.

Multiple operative treatment options exist for acute and chronic AC joint Instabilities, including nonbiological fixation between coracoid and clavicle (e.g., suture loops and synthetic ligaments), biological reconstruction of the CC ligaments (e.g., allograft or autograft tendon reconstruction), ligament and/or tendon transfer (e.g., Weaver–Dunn and Dewar procedures), and fixation with Kirschner wires (e.g., Phemister technique), a hook plate, or other extra-articular techniques [15]. The LockDown device (LockDown Medical, Redditch, Worcestershire, UK) is a synthetic ligament, consisting of a double braided polyethylene terephthalate (Figure 1). Its unique design allows for an anatomic reconstruction of the CC ligaments and restoration of the normal scapular-clavicular relationship. Furthermore, this synthetic ligament may provide adequate stability to allow soft tissue healing [16–18]. However, the LockDown device has not been thoroughly tested in the literature for its ability to provide stability and maintain reduction. Therefore, the purpose of this study was to evaluate the LockDown device utilizing a novel kinematic model along with stress radiographs to evaluate the effects of this implant on AC joint stability and reduction after reconstruction and compare this to its native and severed (injured) states. It was hypothesized that reconstruction with the LockDown device would restore stability and reduction similar to native values; specifically, CC distance would be significantly greater in the severed state compared to the native state but not significantly greater in the reconstructed state compared to the native state. It was also hypothesized that peak AC separation distance in anteroposterior, mediolateral, and inferosuperior directions during shoulder abduction, flexion, and scaption would be significantly different between native and severed states but not significantly different between native and reconstructed states.

## 2. Methods

**Figure 2:**
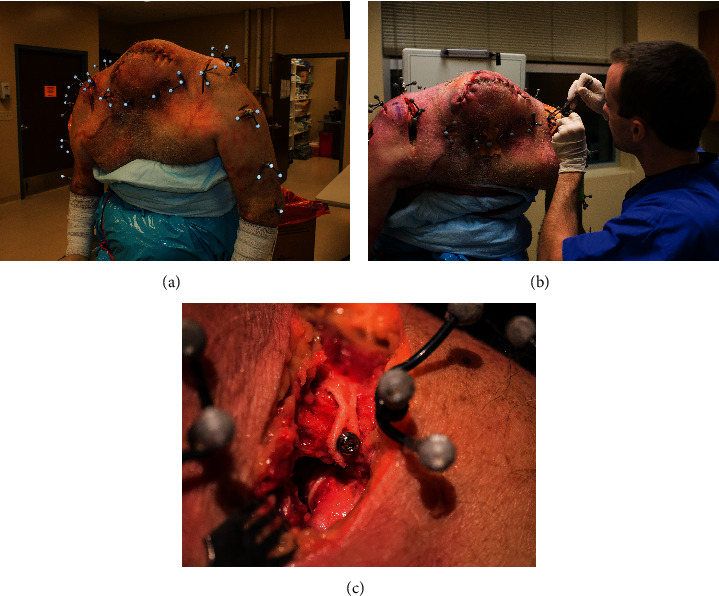
Cadaveric models were used to describe the acromioclavicular distance using biomechanical motion analysis in the “native” (a), “severed” (b), and “reconstructed” (c) states.

Three fresh frozen cadaveric torsos (6 shoulders), with no prior history of relevant medical history for the shoulder, were thawed overnight and mounted upright on a stand allowing for full range of motion of each shoulder. Upon completion of all research, the cadavers were donated to a university. The sample included two men, aged 60 and 70 years, and one woman aged 101 years. Cadaveric models were used to describe the AC distance using biomechanical motion analysis in the “native” (Figure 2(a)), “severed” (Figure 2(b)), and “reconstructed” (Figure 2(c)) states. To prepare the torsos for biomechanical testing, threaded Steimann pins were attached at the medial clavicle, mid-clavicle, lateral clavicle, acromion, superior humerus, mid-humerus, medial and lateral epicondyle of the humerus, inferior scapula, scapular spine, and medial border of scapula. Clusters of three 9.5 mm retroreflective markers (Figure 2(c)) were attached to all bone pins for the purposes of redundant segment tracking, except for the medial clavicle, medial and lateral epicondyles, and the inferior scapular, in which each only had one marker.

Following biomechanical preparation, the shoulders were tested in their “native state,” prior to dissection of the ligaments, using a 10-camera optical motion analysis system (Vicon Nexus, Oxford Metrics, Oxford, UK) to track movement of the retroreflective markers at 240 Hz. Motion analysis was recorded during 3 cycles of glenohumeral motion between approximately 0 and 150 degrees, during separate trials consisting of shoulder flexion, abduction, and scaption. Data were preprocessed using Vicon software (Oxford, UK) and then exported for postprocessing and analysis into Visual 3D (c-motion, Rockville, MD). A 4^th^ order, bidirectional, Butterworth low pass filter with a cut-off frequency of 2 Hz was applied to smooth tracking of the retroreflective marker data. Shoulder flexion and abduction angles were calculated using the formulae and recommendations from the Internal Society of Biomechanics specifically for the shoulder [19]. The absolute distance from the marker on the acromion to the marker on the lateral clavicle was calculated in 3 planes (anteroposterior, mediolateral, and inferosuperior) during the cycles of flexion, abduction, and scaption. The peak, or greatest distance, between the two markers was recorded.

**Figure 3:**
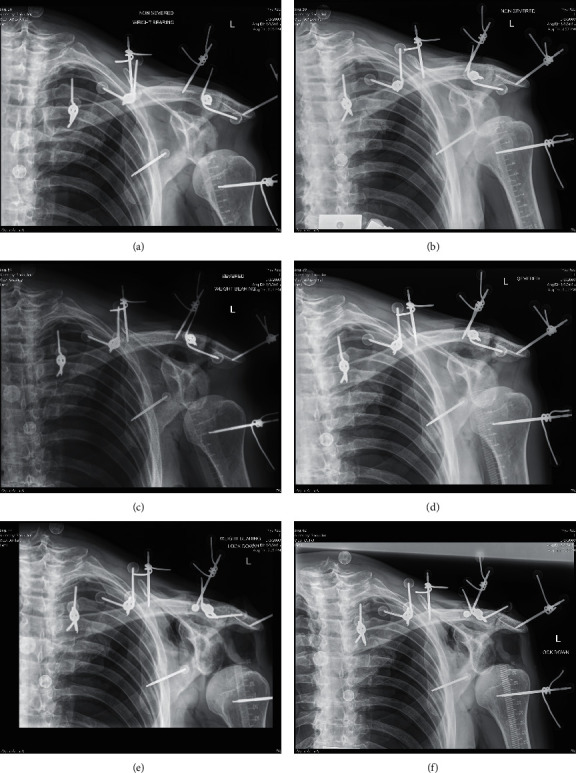
Radiographs were used to measure the coracoclavicular distances in the following states: “native” stressed (a), “native” unstressed (b), “severed” stressed (c), “severed” unstressed (d), “reconstructed” stressed (e), and “reconstructed” unstressed (f).

In addition to biomechanical testing using motion analysis, radiographic testing included standard (unstressed) and weight-bearing (stressed using 4 kg weights hanging from the wrist) Zanca view (Figures 3(a)–3(f)) [20]. The Zanca view was an angled view used to look at the AC joint and the clavicle. A stressed radiograph is used in various joints in orthopedics to assess the stability of the joint. A primarily application of a stressed AC view is to assess for stability in a joint that is suspected to be injured but has normal radiographs, or to assess the degree of instability in a known injury. It is considered positive when it differs from an unstressed view, usually by several millimeters. Radiographic images were evaluated for their CC interval measurement, under both the standard (unstressed) and weight-bearing (stressed) views, using standard clinical techniques.

Following biomechanically and radiographically testing in the “native state” (Figures 2(a), 3(a), and 3(b)), the six shoulders were then biomechanically and radiographically tested in a “severed state” (Figures 2(b), 3(c), and 3(d)), followed by testing in a “reconstructed state” (Figures 2(c), 3(e), and 3(f)). To model the “severed state,” an unstable Grade III injury was simulated by making an anterior incision from the AC joint to the coracoid. The AC joint was exposed as well as the CC interval in the same fashion as during a surgical reconstruction. The AC and CC ligaments were then transected. Finally, the “reconstructed state” was evaluated. Reconstruction was performed using the LockDown device. Consistent with the manufacturer's instructions, the implant was looped around the coracoid and secured over the top of the clavicle with one 3.5 mm cortical screw (Figure 2(c)). The senior surgeon, experienced in AC joint reconstruction, performed all reconstructions. Consistent with the approach to repair an acute Grade III injury, the distal clavicle was not excised.

Statistical analyses were performed using SPSS statistics, version 24 (IBM Corporation, Armonk, New York). Dependent variables included radiographic CC interval measurements in both standard and weight-bearing, as well as biomechanical measures of anteroposterior, mediolateral, and inferosuperior peak AC distances recorded during shoulder flexion, abduction, and scaption for a total of 11 variables under the 3 conditions (native, severed, and reconstructed).

A one-way repeated measure analysis of variance (ANOVA) was used to compare CC distance measurements among the native, severed, and reconstructed states with unstressed and stressed Zanca radiographic views. In addition, a separate two-way repeated measures ANOVA was used to compare peak AC distance measurements in anteroposterior, mediolateral, and inferosuperior directions during shoulder flexion, abduction, and scaption movements among the native, severed, and reconstructed states. Post hoc testing was performed with Bonferroni correction to determine statistical significance between groups. Statistical significance was set at *p* < 0.05.

## 3. Results

**Figure 4:**
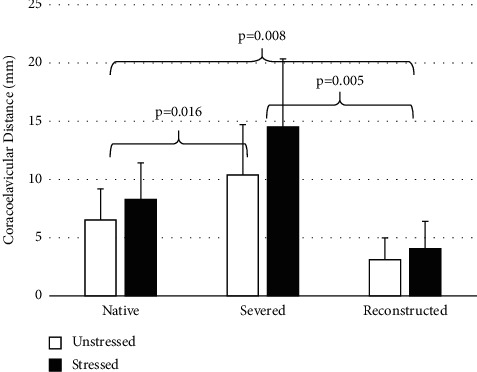
Coracoclavicular distance in the “native,” “severed,” and “reconstructed” states measured using unstressed and stressed zanca views. Brackets and *p*-values indicate statistical significance.

Radiographic analyses indicate that there were main effects for the native, severed, and reconstructed surgical conditions (*p*=0.012) and the Zanca stressed versus unstressed radiographic views (*p*=0.001). CC interval distance was significantly greater (*p*=0.001) across the surgical state in the stressed view compared to the unstressed view. The mean difference between the unstressed and stressed view was 1.8 mm in the native state, 4.1 mm in the severed state, and 0.9 mm in the reconstructed state. CC interval distances in the native, severed, and reconstructed states measured using unstressed and stressed Zanca views demonstrated a consistent pattern for each of the 6 shoulders, with mean (SD) values and significant differences shown in Figure 4. The “native” state (6.5 mm for unstressed and 8.3 mm for stressed) was significantly less (*p*=0.016) compared to the “severed” state (10.4 mm for unstressed and 14.5 mm for stressed), the severed state was significantly greater (*p*=0.005) than the reconstructed state (3.1 mm for unstressed and 4.0 mm for stressed), and the native state was significantly greater (*p*=0.008) than the reconstructed state. CC interval distances decreased from native to reconstructed, an average of 3.3 mm for unstressed and 4.3 mm for stressed.

**Figure 5:**
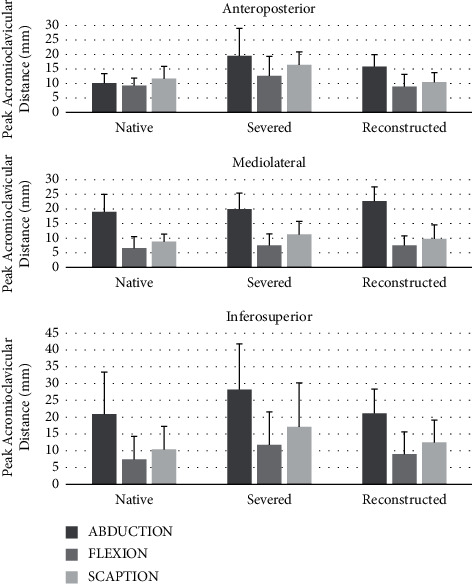
Biomechanical testing of peak acromioclavicular separation distance in anteroposterior, mediolateral, and inferosuperior directions during shoulder abduction, flexion, and scaption for native, severed, and reconstructed states. No significant differences were found in peak acromioclavicular distance among native, severed, and reconstructed conditions during the three movements and three directions.

Biomechanical testing of peak acromioclavicular separation distance in anteroposterior, mediolateral, and inferosuperior directions during shoulder abduction, flexion, and scaption among native, severed, and reconstructed states is shown in Figure 5. Although no significant differences were found among peak AC separation distances, on average, peak AC joint separation during all three motions and movement directions was shown to be restored to 1.5 mm of the native values after reconstruction with LockDown device, which may be clinically relevant.

## 4. Discussion

This study evaluated the biomechanical and radiographic effect of AC joint reconstruction with the LockDown synthetic ligament compared to the native and injured (severed) states. Consistent with our hypothesis, reconstruction with the LockDown device restored AC joint stability and positioning to near native values and therefore appears to be an effective surgical alternative for patients it is appropriate for. Future studies are needed to assess the long-term effects of using the LockDown device on AC joint stability.

The two factors evaluated in the current study were (1) the effect of the LockDown device on AC joint kinematics when compared to the native, severed (injured), and reconstructed states and (2) a radiographic assessment with stressed and unstressed Zanca views [20] to see if the CC interval was restored. The results in the current study demonstrated significantly increased motion in the severed state when compared to the native and reconstructed states. Standard stressed Zanca radiographs [20] showed a significant reduction in the CC interval distance after LockDown when compared to the severed state. Furthermore, the CC interval was shown to be overreduced after reconstruction (4.0 mm) when compared to the native state (8.3 mm), consistent with technique recommendations [17, 21].

Several studies have analyzed various surgical techniques and their biomechanical effect on AC joint reconstruction using the LockDown device. Taranu et al. [21] examined various fixation sites of the LockDown device relative to the conoid tubercle and restoring the acromioclavicular ligament using a modified Neviaser technique and concluded that correct placement of the LockDown device at the conoid tubercle level was critical to allow anatomic joint reduction. Kocsis et al. [17] examined the histology of removed LockDown devices from patients due to failed stabilization and how living tissue reacted to the device and concluded that here are few adverse effects from the implanted LockDown device, and it appears to retain its strength long-term with some limited in-growth into the device. Rashid et al. [2] examined a case study using the LockDown device to enhance AC joint stabilization after failed surgical treatment after acute trauma, and AC stabilization appeared to be restored. Lobao et al. [18] loaded matched-pair cadaveric shoulders cyclically and to failure using either the LockDown device or a coracoclavicular suspensory construct and concluded that the synthetic ligament demonstrated poorer biomechanics than the coracoclavicular suspensory construct, suggesting that a coracoclavicular suspensory construct may be preferable to a synthetic ligament if early rehabilitation was intended.

LaPrade et al. [22] evaluated joint kinematics after reconstruction with the modified Weaver-Dunn technique. They reported significantly greater motion in the cut state than in either the reconstructed or native states. They also noted that the resting position of the distal clavicle postreconstruction sat slightly more anterior and inferior than the native state. Mazzocca et al. [23] compared the modified Weaver-Dunn to a novel arthroscopic CC ligament reconstruction technique and found that their new anatomic technique had significantly less anteroposterior translation and more closely approximated the intact state. Beitzel et al. [24] tested four similar AC reconstruction techniques, all based on a CC reconstruction using two clavicular tunnels and a tendon graft, and found that additional stability was added when the graft end was brought over top of the AC joint and sutured to itself after being passed under the coracoid and through clavicular tunnels. All these aforementioned studies were able to evaluate joint kinematics after various AC surgical reconstruction techniques and compare results to other techniques or to the native joint. However, none of these studies reported the results of stressed and unstressed radiographic evaluation or biomechanical analyses using the LockDown device, which is a unique contribution in the current study.

In order to obtain optimal clinical outcomes, the appropriate treatment should be selected for each patient. Classification systems can aid in decision making, and the ideal classification has a strong clinical correlation. The Rockwood classification, which is the most commonly used amongst clinicians, is based on radiographic findings and, to a degree, helps determine treatment [25]. Grades I and II AC joint injuries are nearly always treated closed, and in grades IV–VI AC joint injuries, the recommendation is usually operative treatment. The optimal treatment of Grade III AC joint injuries has remained controversial; some studies suggest that closed treatment produces acceptable outcomes, while other recent studies have shown operative treatment to be superior [26–28]. In 2014, the International Society of Arthroscopy, Knee Surgery, and Orthopedic Sports Medicine Upper Extremity committee determined that the Rockwood classification did not adequately guide treatment decisions for Grade III injuries and broke these into Grades IIIA and IIIB, determined by clavicular stability on cross arm adduction radiograph, as well as absence or presence of significant scapular dyskinesia (Table 1) [29]. They concluded that the unstable Grade IIIB injury may benefit from operative intervention. More recently, Gorbaty et al. [30] provided the most up-to-date Rockwood classification of acromioclavicular joint separations (Table 1).

The ideal system for treating unstable AC joint injuries would provide enough stability to allow for soft tissue healing while restoring AC joint position and normal joint biomechanics. Providing stability in the anteroposterior and inferosuperior planes is vital in order to restore normal motion between the clavicle and the shoulder girdle. Additional studies are needed to assess the long-term effects of the LockDown device in providing stability in anteroposterior and inferosuperior planes during abduction, flexion, and scaption movements.

With complete AC joint ligament disruption, as in unstable Grade III and Grades IV–VI, the clavicle is dissociated from the acromion, thus destabilizing the shoulder girdle and allowing it to drop inferiorly. This lack of stability and maligned positioning can lead to chronic pain and dysfunction [3, 31]. Another potential sequelae of AC joint injury are scapular dyskinesis, which has been shown to occur in up to 70% of chronic dislocations [32]. A combination of painful range of motion and a lack of normal clavicular-scapula coupling can result in inhibition and disorganization of recruitment patterns in stabilizing muscles [32]. This results in abnormal movement and function of the scapula and may lead to chronic pain and contribute to scapulohumeral functional deficits with motion. Unstable AC joint injuries have also been shown to result in excessive motion at the destabilized distal clavicle, potentially resulting in early AC joint arthritis [31].

Strength of the current study was the addition of both stressed and unstressed radiographs and three-dimensional kinematic analyses. This allowed the assessment of the CC interval and the placement of the implant. In addition, obtaining radiographs with motion pins in place allowed for construction of a more accurate kinematic model. In contrast to previous biomechanical studies, intact torsos were used, which decreased the potential compromise of the sternoclavicular, scapulothoracic, and glenohumeral joint motions. Finally, three-dimensional motions allowed the assessment of AC joint stability in flexion, abduction, and scaption motions and in anteroposterior, mediolateral, and inferosuperior directions.

There are limitations in the current study. Firstly, being a cadaveric study, the in vivo forces, kinematics, and muscular contractions cannot be fully replicated. This also does not allow for progressive soft tissue healing, the outcome of which is the ultimate goal of any reconstruction technique. There is no way to determine outcomes related to soft tissue healing around the artificial ligaments using the current procedure, or how the CC interval is maintained in an in vivo model [33–36]. Further research addressing this topic should be considered. Secondly, the sample size was relatively small, with only 6 total shoulders, which may have prevented observing significant differences in peak AC separations in the three motions and three directions that were assessed. Nevertheless, there was a trend that demonstrated an increase in peak AC separation going from the native state to the severed state, and then a decrease in peak AC separation going from the severed state to the reconstructed state that approached native values in the flexion, abduction, and scaption motions for both the anteroposterior and inferosuperior directions.

## 5. Conclusion

The LockDown synthetic ligament is an anatomic coracoclavicular ligament used for reconstruction of unstable AC joints that sustain a Grade III AC injury or higher. The LockDown device is designed to provide adequate stability while promoting soft tissue healing. Employing the LockDown device restored joint separation to near native states throughout all planes of motion. It also decreased excursion in all planes when compared to the injured state. Finally, the LockDown significantly reduced the widened CC interval seen on stress radiographs with ligamentous instability.

## Figures and Tables

**Table 1:** Rockwood and ISAKOS classifications for AC joint injury.

Classification	AC ligaments	CC ligaments	Deltoid and trapezius	Radiographic hallmark of AC joint and CC interval	Treatment
Rockwood I	Sprained	Intact	Intact	Intact but joint space may be may widened	Nonoperative; 6–12 weeks of rehabilitation
Rockwood II	Complete tear	Sprained	Possible partial detachment	CC interval <25% increase and disrupted AC joint	Nonoperative; 6–12 weeks of rehabilitation
Rockwood III	Complete tear	Disrupted	Likely detached from lateral clavicle	CC interval 25–100% increase	Controversial–usually non-op initially
ISAKOS IIIA	Complete tear	Disrupted	Likely detached from lateral clavicle	Clavicle not overriding on adduction view	Favors nonoperative
ISAKOS IIIB	Complete tear	Disrupted	Likely detached from lateral clavicle	Clavicle overriding on adduction view	Favors surgery
Rockwood IV	Complete disruption	Partial or complete disruption	Likely detached from lateral clavicle	AC joint dislocated; clavicle posterior into or through trapezius on axillary view	Surgery
Rockwood V	Complete disruption	Complete disruption	Likely detached from lateral clavicle	AC joint dislocated; extreme vertical incongruity between lateral clavicle and acromion; CC interval 100% to 300% increase	Surgery
Rockwood VI	Complete disruption	Intact and interval is decreased or reversed^*∗*^	Intact, partial, or complete detachment	AC joint dislocated; lateral clavicle displaced inferior to acromion and found in subacromial or subcoracoid space	Surgery

^*∗*^In a continuation of type VI, the clavicle is displaced inferior to the coracoid process, and the CC ligaments are completely torn.

## Data Availability

All the data are directly available by contacting the lead author.
